# Comparing a PD-L1 inhibitor plus chemotherapy to chemotherapy alone in neoadjuvant therapy for locally advanced ESCC: a randomized Phase II clinical trial: A randomized clinical trial of neoadjuvant therapy for ESCC

**DOI:** 10.1186/s12916-023-02804-y

**Published:** 2023-03-08

**Authors:** Yong Li, Aiping Zhou, Shuoyan Liu, Ming He, Keneng Chen, Ziqiang Tian, Yin Li, Jianjun Qin, Zhen Wang, Haiquan Chen, Hui Tian, Yue Yu, Wang Qu, Liyan Xue, Shun He, Shuhang Wang, Fenglong Bie, Guangyu Bai, Bolun Zhou, Zhaoyang Yang, Huiyao Huang, Yan Fang, Benjamin Li, Xiangrong Dai, Shugeng Gao, Jie He

**Affiliations:** 1 Department of Thoracic Surgery, National Cancer Center/National Clinical Research Center for Cancer/Cancer Hospital, Chinese Academy of Medical Sciences and Peking Union Medical College, NO. 17, Panjiayuannanli, Chaoyang District, Beijing, 100021 China; 2 Department of Medical Oncology, National Cancer Center/National Clinical Research Center for Cancer/Cancer Hospital, Chinese Academy of Medical Sciences and Peking Union Medical College, Beijing, 100021 China; 3 Fujian Provincial Cancer Hospital, Fujian, China; 4 The Fourth Hospital of Hebei Medical University, Hebei, China; 5 Peking University Cancer Hospital, Beijing, China; 6 Fudan University Cancer Hospital, Shanghai, China; 7 Qilu Hospital of Shandong University, Shandong, China; 8 Clinical Trial Center, National Cancer Center/National Clinical Research Center for Cancer/Cancer Hospital, Chinese Academy of Medical Sciences and Peking Union Medical College, Beijing, 100021 China; 9 Department of Pathology, National Cancer Center/National Clinical Research Center for Cancer/Cancer Hospital, Chinese Academy of Medical Sciences and Peking Union Medical College, Beijing, 100021 China; 10 Department of Endoscopy, National Cancer Center/National Clinical Research Center for Cancer/Cancer Hospital, Chinese Academy of Medical Sciences and Peking Union Medical College, Beijing, 100021 China; 11 PET-CT Center, National Cancer Center/National Clinical Research Center for Cancer/Cancer Hospital, Chinese Academy of Medical Sciences and Peking Union Medical College, Beijing, 100021 China; 12 Lee’s Pharmaceutical Limited, Shenzhen, China

**Keywords:** PD-L1, Chemotherapy, Neoadjuvant treatment, Esophageal squamous cell carcinoma (ESCC), Major pathological response (MPR)

## Abstract

**Background:**

A Phase II study was undertaken to evaluate the safety and efficacy of the neoadjuvant socazolimab, a novel PD-L1 inhibitor, in combination with nab-paclitaxel and cisplatin for locally advanced esophageal squamous cell carcinoma (ESCC).

**Methods:**

Sixty-four patients were randomly divided between the Socazolimab + nab-paclitaxel + cisplatin (TP) arm (*n* = 32) and the control arm (*n* = 32), receiving either socazolimab (5 mg/kg intravenously (IV), day 1) or a placebo with nab-paclitaxel (125 mg/m^2^ IV, day 1/8) and cisplatin (75 mg/m^2^ IV, day 1) repeated every 21 days for four cycles before surgery. The primary endpoint was major pathological response (MPR), and the secondary endpoints were pathological complete response (pCR), R0 resection rate, event-free survival (EFS), overall survival (OS), and safety.

**Results:**

A total of 29 (90.6%) patients in each arm underwent surgery, and 29 (100%) and 28 (98.6%) patients underwent R0 resection in the Socazolimab + TP and Placebo + TP arms, respectively. The MPR rates were 69.0 and 62.1% (95% Confidence Interval (CI): 49.1–84.0% vs. 42.4–78.7%, *P* = 0.509), and the pCR rates were 41.4 and 27.6% (95% CI: 24.1–60.9% vs. 13.5–47.5%, *P* = 0.311) in the Socazolimab + TP and Placebo + TP arms, respectively. Significantly higher incidence rates of ypT0 (37.9% vs. 3.5%; *P* = 0.001) and T downstaging were observed in the Socazolimab + TP arm than in the Placebo + TP arm. The EFS and OS outcomes were not mature.

**Conclusions:**

The neoadjuvant socazolimab combined with chemotherapy demonstrated promising MPR and pCR rates and significant T downstaging in locally advanced ESCC without increasing surgical complication rates.

**Trial registration:**

Registration name (on clinicaltrials.gov): A Study of Anti-PD-L1 Antibody in Neoadjuvant Chemotherapy of Esophageal Squamous Cell Carcinoma. Registration number: NCT04460066.

**Supplementary Information:**

The online version contains supplementary material available at 10.1186/s12916-023-02804-y.

## Statement of translational relevance

This study is the first multicenter (six high-volume medical centers in China), randomized, double-blind, placebo-controlled study of anti-PD-L1 antibodies combined with chemotherapy for locally advanced ESCC. Additionally, this study is the first to test a PD-L1 inhibitor in the context of neoadjuvant treatment. Socazolimab in combination with albumin-bound paclitaxel and cisplatin revealed outstanding MPR and pCR rates, which were higher than those of albumin-bound paclitaxel and cisplatin alone. A significantly higher frequency of ypT0 was observed in the experimental group (37.9%) than in the control group (3.4%), which may reduce the local recurrence for patients with locally advanced ESCC. The favorable efficacy and safety data indicated that albumin-bound paclitaxel and cisplatin could be recommended as a preferred accompaniment for immune checkpoint inhibitors in neoadjuvant treatment for locally advanced ESCC. Our study provides a meaningful reference dataset for the design of future Phase III studies of neoadjuvant therapy for locally advanced ESCC.

## Background

A total of 604,000 new cases of esophageal cancer and 544,000 deaths from this cause were reported globally in 2020, placing esophageal cancer 7th in incidence and 6th in mortality among all cancer types [1]. The incidence and histology of esophageal squamous cell carcinoma (ESCC) are related to geographic location, accounting for the fact that 90% of esophageal cancer cases occur in Asia. The prognosis for esophageal cancer is poor, with a 5-year survival rate of less than 20%, falling to 5% for advanced cases [2].

Surgery-based combination therapy is currently the primary treatment for nonmetastatic ESCC. Neoadjuvant concurrent chemoradiotherapy is an essential part of preoperative treatment that can greatly improve R0 resection and survival rates [3, 4]. Based on the results of the JCOG1109 trial, patients receiving neoadjuvant chemotherapy achieved satisfactory prognoses (3-year overall survival (OS): 57–62.6%), and the pathological complete response (pCR) rates of neoadjuvant chemotherapy were significantly lower than those of concurrent chemoradiotherapy in patients with ESCC [5–7].

Recently, programmed cell death protein 1 (PD-1) inhibitors have been shown to exert antitumor effects in various tumor types [8] and have also demonstrated remarkable performance in treating advanced ESCC. In the KEYNOTE-590 study, pembrolizumab (a PD-1 inhibitor) combined with chemotherapy significantly prolonged OS compared to a placebo-chemotherapy treatment in the first-line treatment of patients with advanced ESCC (median survival: 12.6 months vs. 9.8 months; hazard ratio (HR) 0.72, 95% Confidence Interval (CI) 0.60–0.88), with tolerable toxicity [9]. Additionally, the therapeutic strategy of chemoimmunotherapy has been investigated for the neoadjuvant treatment of resectable locally advanced ESCC, with encouraging results, and several Phase II clinical studies have demonstrated that combining neoadjuvant treatment using PD-1 inhibitors with chemotherapy induces pCR rates ranging from 16.7–45% [10–16]. Current applications of immune checkpoint inhibitors for treating ESCC are largely dominated by PD-1 inhibitors, whereas relatively little attention has been focused on programmed cell death ligand 1 (PD-L1) inhibitors. In one recent Phase II study, it was shown that the use of a PD-L1 inhibitor-chemotherapy combination as the first-line treatment for patients with unresectable locally advanced ESCC resulted in impressive median OS (11.6 months) and overall response rate (ORR; 52.2%) values, an indication of the potential therapeutic efficacy of PD-L1 inhibitors for ESCC treatment [17].

Socazolimab, a novel humanized IgG1 monoclonal antibody against PD-L1, has been tested in several clinical trials, including trials in small-cell lung cancer and cervical cancer, with promising results [18]. Based on the results of our previous study of neoadjuvant chemotherapy [19], we designed a multicenter, randomized, double-blind Phase II study to assess the feasibility, safety, and efficacy of the neoadjuvant socazolimab plus chemotherapy followed by quality-control minimally invasive esophagectomy (McKeown) in patients with resectable locally advanced ESCC.

## Methods

### Study design

Our study consisted of two phases; the first phase was an exploratory Phase IB component concentrating on safety, and the second consisted of a multicenter, randomized, double-blind Phase II assessment. Our research was conducted at six hospitals, including the Cancer Hospital Chinese Academy of Medical Sciences, and was approved by the ethics committees of all participating institutes (registration number NCT04460066 on clinicaltrials.gov).

### Participants and procedures

This clinical trial was exploratory in nature, aiming to investigate the safety and efficacy of socazolimab, and a total of 70 patients (Phase Ib: 6 patients; Phase II: 64 patients) were enrolled based on previous experience. Patients who had histologically or cytologically confirmed ESCC clinically staged as T2N + M0 or T3-4aN ± M0 before treatment and who had no history of antitumor therapy, were 18–75 years old, had an Eastern Cooperative Oncology Group (ECOG) score of 0–1, and could tolerate chemotherapy were eligible for inclusion in our analysis. We excluded ESCC patients who had cervical or combined cervical, supraclavicular, abdominal, retroperitoneal, and pelvic lymph node metastasis; had interstitial lung disease or other preexisting active, possibly recurrent autoimmune disease; or had been treated with corticosteroids (> 10 mg/d prednisone or equivalent) or other immunosuppressants within 2 weeks before the first drug administration. The full eligibility criteria are provided in the study protocol (see Appendix). All patients provided written informed consent prior to enrollment in the study.

Sixty-four patients in the Phase II study were randomly divided in a 1:1 ratio between the experimental (Socazolimab + nab-paclitaxel + cisplatin (TP)) and control (Placebo + TP) groups and received four cycles of TP combined with socazolimab or placebo, depending on which group they were in. Socazolimab or placebo was administered to patients intravenously (IV) at a dose of 5 mg/kg at 1 d; nab-paclitaxel was administered at 125 mg/m^2^ IV at 1 d and 8 d; and cisplatin was administered at 75 mg/m^2^ IV at 1 d. All drugs were administered every 3 weeks for four cycles.

All patients underwent video-assisted thoracoscopy esophagectomy (McKeown) 4–6 weeks after neoadjuvant treatment. Two-field lymphadenectomy was performed, including total mediastinal lymph node dissection and bilateral para-laryngeal recurrent nerve lymph node and celiac lymph node dissection. The stomach was dissociated laparoscopically to create a tubular stomach, which was anastomosed through the left neck of the esophageal bed.

Enrolled patients were unblinded after the surgery. Five to six weeks postoperatively, adjuvant therapy with socazolimab for 12 cycles or 9 months (whichever came first) was administered to patients in the Socazolimab + TP group who underwent R0 resection but did not achieve pCR, while no further postoperative therapy was administered to patients with R0 resection in the Placebo + TP group (according to the National Comprehensive Cancer Network (NCCN) guidelines). Treatment was discontinued in the event of disease progression, intolerable adverse events, or the investigators' judgment that the risks outweighed the benefits or withdrawal of informed consent.

The clinical stages of all participants were determined via cervicothoracic enhanced computer tomography (CT) and ultrasound endoscopy, and 18F-fluoro-2-deoxy-d-glucose (18F-FDG) positron emission tomography (PET)-CT was also performed before and after neoadjuvant treatment. We evaluated the efficacy of neoadjuvant therapy every 2 weeks prior to surgery based on the RECIST v1.1 standard supplemented by the iRECIST standard. Postoperative examinations were conducted at 1 month and then every 3 months following surgery. The reference for adverse events was based on CTCAE5.0 standards.

### Randomization

Block randomization (block length of four, SAS 9.4 software) was used for the analysis, with participants assigned to either the Socazolimab + TP or Placebo + TP treatment groups. Identical packages of socazolimab/placebo were prepared and numbered by the sponsor, and the relevant drug package was randomly assigned to individuals within the appropriate groups in a 1:1 ratio (in accordance with the SAS 9.4 PROC PLAN procedure). All clinical staff involved in drug allocation were blinded to the study, as were the sponsors, investigators, patients, clinical contract research organizations, and independent review committees. Patient randomization and assignment of clinical trial drugs followed Interactive Web Response System-Balance (Medidata Solutions) protocols. Preparation and administration of nab-paclitaxel and cisplatin were conducted in accordance with the manufacturers’ instructions.

### Endpoint

The primary endpoint of the Phase Ib study was dose-limiting toxicity (DLT), with the intention that a Phase II study would be conducted if DLT occurred in fewer than two-sixths of the treated patients, whereas the study would be terminated if DLT occurred in two or more patients. DLT was defined as any of the following adverse events occurring within 21 d of initial drug administration: Grade 4 neutropenia > 7 d; ≥ Grade 3 neutropenia with fever (T ≥ 38.5 °C) lasting > 24 h; Grade 4 thrombocytopenia or Grade 3 thrombocytopenia with bleeding; Grade 4 anemia; ≥ Grade 3 clinically significant nonhematologic toxicity; ≥ Grade 2 immune-related cardiotoxicity, immune-related pneumonia, immune-related ophthalmopathy; and ≥ Grade 3 other immune-related toxicity.

The primary endpoint of the Phase II study was the major pathological response (MPR) rate, with secondary endpoints consisting of the R0 resection rate, pCR rate, safety, disease-free survival (DFS), event-free survival (EFS), and OS. The Becker standard was used to evaluate pathological regression of the primary tumor after surgery. No residual tumor cells were defined as type 1a, less than 10% were defined as type 1b, 10–50% were defined as type 2, and the remainder were defined as type 3. Pathological remission assessed at Grades 1a and 1b was considered to be MPR (including pCR), while pCR was defined as the absence of residual tumor cells (including primary tumors and lymph nodes).

### Downstaging

The data regarding downstaging were compared between the Socazolimab + TP and Placebo + TP groups. Patients with T downstaging or N downstaging were selected separately for further comparison. T0, Tis/T1, T2, T3, and T4 were defined as Grades 0, 1, 2, 3, and 4, respectively, and downstaging grades were quantitatively calculated. For example, the T downstaging grade is 3 for a patient whose tumor was evaluated as T3 (i.e., Grade 3) prior to surgery and T0 (i.e., Grade 0) after surgery. The grades of T downstaging in the Socazolimab + TP group and Placebo + TP group were then compared, and statistical analysis was performed.

### Circulating tumor DNA (ctDNA) mutation detection

In this clinical study, we collected biopsy tissue and blood samples from 37 patients (52.9% of all participants) at the Cancer Hospital Chinese Academy of Medical Sciences center. Tumor biopsy tissue samples were collected before neoadjuvant treatment by esophagogastroscopy. Peripheral blood samples were collected at pretreatment (baseline, C1D1, the first day of the first cycle before administration), the end of the first neoadjuvant treatment cycle (C2D1, the first day of the second cycle before administration), the end of the second cycle (C3D1), the end of the third cycle (C4D1), and the end of the fourth cycle (one day before the operation, preO). Tumor-specific mutations were identified from exome sequencing of tumor biopsy tissue-matched white blood cells. On average, 28 (7–50) mutations per patient were selected for the detection of ctDNA in plasma samples using a personalized assay based on Mutation Capsule, a mutation profiling technology [20]. The ctDNA fraction was calculated based on the number and fraction of the mutations detected in plasma samples to determine the fraction of ctDNA among cell-free DNA (cfDNA) [21]. The detailed methods of ctDNA detection were shown in Additional File 1 [20–22]. We focused on ctDNA clearance before the operation, which means that the ctDNA fraction was zero at preO. According to the ctDNA fraction before the operation, the patients were divided into ctDNA-positive (ctDNA +) and ctDNA-negative (ctDNA-) groups.

### Statistical analysis

SAS 9.4 was used for all statistical analyses. Measurement data were expressed as the means and standard deviations, and *t* tests were used for comparisons between the two treatment groups. A Kruskal‒Wallis rank test was used for nonnormally distributed data. Count data were expressed as frequencies (percentages), and confidence intervals were calculated based on a normal distribution approximation using the Clopper-Pearson method. The confidence intervals of proportions were calculated using the Wilson procedure with a correction for continuity. A chi-square test or Fisher's exact test was used for comparisons between the two groups. All statistical tests were two-sided, and statistical significance was set at *P* < 0.05.

## Results

### Baseline characteristics

**Fig. 1:**
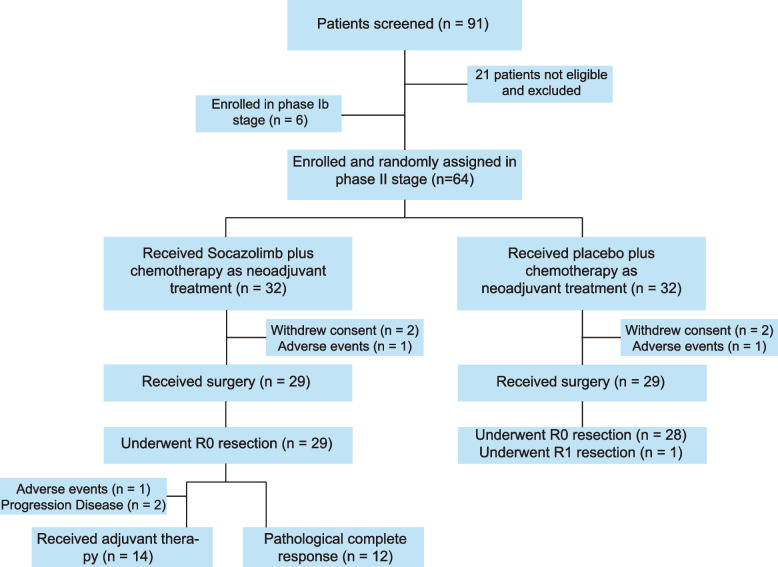
Study flowchart

A total of 70 ESCC patients were enrolled in the study from February 9, 2021, to July 15, 2021, including the six patients in the Phase IB study. The 64 patients enrolled in the Phase II study were equally divided into the Socazolimab + TP or the Placebo + TP arms (see flowchart in Fig. 1). The characteristics of the patients included in the Phase IB study are presented in Additional File 2: Table S1, and those of the patients included in the Phase II study are presented in Table 1. The median age of patients in the Phase II study was 62 years (47–74 years), with males accounting for 79.69% of patients. The clinical stages of the patients are shown in Table 1, and 39 patients (60.94%) were diagnosed with stage III disease. No significant differences in clinical characteristics were found between the Socazolimab + TP and Placebo + TP groups.

**Table 1:** Baseline characteristics of the intention-to-treat population

Characteristic	Socazolimab + TP (*n* = 32)	Placebo + TP (*n* = 32)	*P*
Age, years			0.388
Median (range)	61.0 (53–72)	63.0 (47–74)	
Sex			0.120
Male	23 (71.9)	28 (87.5)	
Female	9 (28.1)	4 (12.5)	
BMI, kg/m^2^			0.381
Median (range)	23.4 (13.7–30.6)	23.5 (19.1–28.5)	
ECOG PS			0.522
0	25 (78.1)	27 (84.4)	
1	7 (21.9)	5 (15.6)	
Tumor location			0.454
Proximal third	9 (28.1)	5(15.6)	
Middle third	18 (56.3)	20 (62.5)	
Distal third	5 (15.6)	7 (21.9)	
Clinical T stage			1.000
cT2	5 (15.6)	4 (12.5)	
cT3	26 (81.3)	27 (84.4)	
cT4a	1 (3.1)	1 (3.1)	
Clinical N stage			0.514
cN0	6 (18.8)	7 (21.9)	
cN1	17 (53.1)	12 (37.5)	
cN2	9 (28.1)	12 (37.5)	
cN3	0 (0.0)	1 (3.1)	
Clinical stage			0.756
II	11 (34.4)	8 (25.0)	
III	19 (59.4)	20 (62.5)	
IVA	1 (3.1)	2 (6.3)	

Data are presented as No. (%)

*Abbreviations*: *TP* nab-paclitaxel + cisplatin, *BMI* body mass index, *ECOG* Eastern Cooperative Oncology Group performance status

### Patient outcomes

No DLT events occurred during the first cycle of treatment in the Phase IB component. In the phase II component, 64 patients received four cycles of neoadjuvant therapy, and as of January 5, 2022, 58 patients had undergone surgery. In the Socazolimab + TP group, surgery was not performed for three patients after neoadjuvant therapy (refusal to perform surgery for two patients and grade 3 thrombocytopenia emerged in one patient). In the Placebo + TP group, surgery was not performed on three patients after neoadjuvant therapy (two patients refused to undergo surgery, and intestinal obstruction occurred in one patient). In the Socazolimab + TP and Placebo + TP groups, 28 (87.5%) and 29 (90.6%) patients completed the treatment for more than or equal to three cycles, respectively, of which 24 (75.0%) and 27 (84.4%) completed treatment for four cycles, respectively. The median number of treatment cycles in both groups was four (range 1–4). In the Socazolimab + TP group, 14 patients continued adjuvant therapy with socazolimab after surgery, and one patient withdrew informed consent. As of January 5, 2022, 13 patients continued to receive adjuvant therapy. There are currently 52 patients (74.3%, other patients withdrew from the clinical trial) undergoing postoperative follow-up. One patient in the Placebo + TP group died due to postoperative disease progression, and one patient in the Socazolimab + TP group developed brain metastases after surgery.

**Fig. 2:**
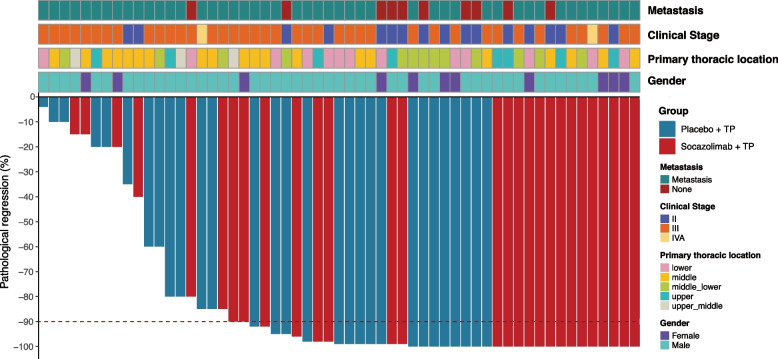
Comparison of pathologic regression between the Socazolimab + TP and Placebo + TP groups (TP: nab-paclitaxel/cisplatin). The upper plot shows the clinicopathological information of each patient. The lower plot shows the pathological regression of the neoadjuvant treatment. Different colors represent different groups of this study

Of patients who underwent surgery, 29 (100.0%) in the Socazolimab + TP group and 28 (96.6%) in the Placebo + TP group reached R0, and 20 (69.0%, 95% CI: 49.1–84.0%) and 18 (62.1%, 95% CI: 42.4–78.7%) cases reached MPR (*P* = 0.581), respectively. The proportion of primary tumors at the ypT0 stage in the Socazolimab + TP group was considerably higher than that in the Placebo + TP group (37.9% vs. 3.5%; *P* = 0.001). In addition, 12 patients (41.4%, 95% CI: 24.1–60.9%) in the Socazolimab + TP group achieved pCR (ypT0/TisN0M0) compared to 8 patients (27.6%, 95% CI: 13.5–47.5%) in the Placebo + TP group. The postoperative pathological response results of all patients who underwent surgery are shown in Table 2 (full Phase Ib data are presented in Additional File 2: Table S2) and Fig. 2. Mature DFS, EFS, and OS rates were not observed.

**Table 2:** Distribution of pathologic stage groups after surgery

	Socazolimab + TP (*n* = 29)	Placebo + TP (*n* = 29)
Pathologic T Stage		
T0	11 (37.9)	1 (3.4)
Tis/T1	5 (17.2)	13 (44.8)
T2	5 (17.2)	4 (13.8)
T3	8 (27.6)	11 (37.9)
Pathologic N Stage		
N0	20 (69.0)	21 (72.4)
N1	7 (24.1)	3 (10.3)
N2	1 (3.4)	4 (13.8)
N3	1 (3.4)	1 (3.4)
Pathologic Stage		
I	17 (58.6)	17 (58.6)
II	3 (10.3)	4 (13.8)
IIIA	4 (13.8)	0 (0.0)
IIIB	4 (13.8)	7 (24.1)
IVA	1 (3.6)	1 (3.4)

Data are presented as No. (%)

*Abbreviations*: *TP* nab-paclitaxel + cisplatin

### Downstaging

**Fig. 3:**
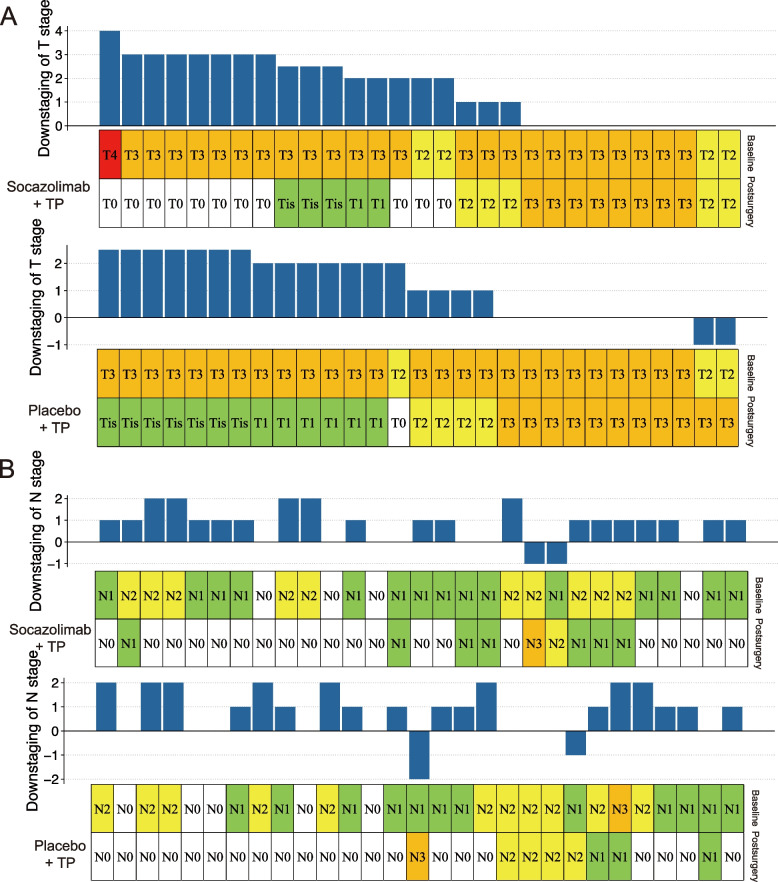
Downstaging of T stage (A) and N stage (B) in the Socazolimab + TP and Placebo + TP groups after neoadjuvant treatment (TP: nab-paclitaxel/cisplatin). The upper plot shows the exact number of downstaging of T or N stage. The lower plot shows the T or N stage of each patient before the neoadjuvant treatment and after the surgery. Each column represents a patient enrolled in this study

Only the pathological T stage of the Socazolimab + TP group differed significantly from that of the Placebo + TP group; no significant differences in the pathological N stage were detected between the two groups. Data for changes in stage are presented in Fig. 3. Downstaging of T stage occurred in 19 (65.5%) and 18 (62.1%) patients in the Socazolimab + TP or Placebo + TP groups, respectively. Two (6.9%) patients in the Placebo + TP group exhibited an increasing T stage (Additional File 2: Table S3). T downstaging of Grades 3–4 was observed in eight (42.1%) and zero (0.0%) patients in the Socazolimab + TP and Placebo + TP groups, respectively (Additional File 2: Table S4). Of the patients who experienced T downstaging, 11 (57.9%) in the Socazolimab + TP group and one (5.6%) in the Placebo + TP group had T0 stage tumors (Additional File 2: Table S4).

### PET/CT

Fifty-three patients (25 in the Socazolimab + TP group and 28 in the Placebo + TP group) underwent PET/CT twice before and after neoadjuvant treatment. The mean maximum standardized uptake value (SUVmax) of the primary tumors was 16.4/16.1 (Socazolimab + TP group/Placebo + TP group) before treatment but fell to 4.9/4.4 (Socazolimab + TP group/Placebo + TP group) after neoadjuvant therapy; both of these decreases were significant (*P* < 0.001). Consequently, the mean decreased ratio of SUVmax (SUVmax-DR) during chemotherapy was 70.4%/69.2% (Socazolimab + TP group/Placebo + TP group). Patients were divided into subgroups that showed response (including pCR and MPR, 18 with Socazolimab + TP group, and 18 with Placebo + TP group) and nonresponse (7 with Socazolimab + TP group, and 10 with Placebo + TP group) to neoadjuvant therapy. The area under the curve (AUC) for receiver operating characteristic (ROC) analysis was 0.736/0.872 (Socazolimab + TP group/Placebo + TP group) for SUVmax-DR. At an SUVmax-DR cutoff of 74.0%, the sensitivity, specificity, positive, and negative predictive values for predicting pathological major response were 80.6, 83.4, 90.6, and 66.7%, respectively. The results of our analysis indicated that, for esophageal cancer, the change rate of PET/CT metabolic parameters before and after neoadjuvant chemotherapy (SUVmax-DR) had good predictive value for the efficacy of neoadjuvant chemotherapy (Additional File 3: Fig. S1). However, neoadjuvant therapy caused inflammatory changes in some patients, leading to false-positive results (Additional File 3: Fig. S1).

### ctDNA mutation analysis

The ctDNA fraction gradually decreased as the neoadjuvant treatment progressed (Additional File 3: Fig. S2). ctDNA could be cleared in some patients who respond well to neoadjuvant treatment. In the Socazolimab + TP group (*n* = 19), 7 patients (36.8%) achieved ctDNA clearance (Additional File 3: Fig. S2A). In the Placebo + TP group (*n* = 18), 4 patients (22.2%) achieved ctDNA clearance (Additional File 3: Fig. S2B). However, there was no statistically significant difference between the two groups (*P* = 0.476, Additional File 3: Fig. S2C).

### Surgical indicators and complications

The median intervals from the final drug administration to surgery in the Socazolimab + TP and Placebo + TP groups were 47 (28–127) and 45 (31–67) days, respectively (*P* = 0.196), and the median numbers of dissected lymph nodes were 44 (10–69) and 35 (16–99), respectively. In the Socazolimab + TP treatment group, one (3.4%) patient had both anastomotic leakage and bile duct obstruction, and one (3.4%) contracted pneumonia. In the Placebo + TP group, one (3.4%) patient had pneumothorax, and one (3.4%) patient died from pneumonia. No significant differences were observed in the length of hospital stay, duration of surgery, intraoperative bleeding volume, number of lymph nodes dissected, time from the last neoadjuvant therapy administration to surgery, or surgical complications between the two groups (*P* > 0.05) (Additional File 2: Table S5).

### Safety

Hematological toxicity and gastrointestinal events were the most common adverse events (AEs). AEs of Grade ≥ 3 occurred in 65.6 and 62.5% of patients in the Socazolimab + TP and Placebo + TP groups, respectively. The most common AEs included neutropenia (59.4% vs. 56.3% of the two groups, respectively), leukopenia (43.8% vs. 25.0%), hypokalemia (18.8% vs. 0), anemia (12.5% vs. 6.3%), and decreased platelet count (12.5% vs. 6.3%) (Additional File 2: Table S6; data for Phase IB are presented in Additional File 2: Table S7). Immune-related AEs (irAEs) occurred in 25.0 and 9.4% of patients in the two groups, respectively, with increased thyroxine being the most common (6.3% vs. 0) (Additional File 2: Table S8). Treatment-related severe AEs (SAEs) were observed in 28.1 and 12.5% of patients in the two groups, respectively, and SAEs that emerged during neoadjuvant therapy were observed in 25.0 and 6.3% of patients in the two groups, respectively (Additional File 2: Table S9).

Dosage was reduced for eight patients (25.0%) due to AEs arising during neoadjuvant therapy. The dose reductions of nab-paclitaxel in the Socazolimab + TP and Placebo + TP groups were 15.6 and 18.8%, respectively, and those of cisplatin were 6.3 and 3.1%, respectively. Treatment was terminated for two patients (6.3%) in the Socazolimab + TP group due to a urinary tract infection and an acute kidney injury, while a single patient (3.1%) in the Placebo + TP group was discharged due to an intestinal obstruction.

## Discussion

In the first Phase II randomized controlled study of PD-L1 inhibitor plus chemotherapy in the neoadjuvant treatment of locally advanced ESCC, treatment with socazolimab in combination with nab-paclitaxel and cisplatin demonstrated a better antitumor effect, with an MPR rate of 69.0% at the primary tumor site and an increase of 6.9% compared to chemotherapy alone, although there was no significant difference. This result is comparable to the MPR rate of 63.4% resulting from concurrent chemoradiotherapy with paclitaxel and cisplatin reported in a recently reported Phase III randomized controlled study [7]. Further analysis revealed that the proportion of patients achieving complete regression of the primary tumor (ypT0) in the Socazolimab + TP group was considerably higher than that in the Placebo + TP group, indicating that the PD-L1 inhibitor plus chemotherapy approach enhanced complete regression of the primary tumor. Furthermore, the Socazolimab + TP group showed a pCR rate of 41.4%, a level 13.8% higher than the Placebo + TP group. Moreover, the pCR rate of the chemoimmunotherapy treatment presented here seemed numerically higher than that reported for cisplatin coupled with fluorouracil (2.6%) or paclitaxel (2.9%) in previous Phase III trials of neoadjuvant chemotherapy, which is relatively low [5, 7]. This result suggests that Socazolimab + TP is a promising neoadjuvant strategy and warrants further investigation.

Currently, neoadjuvant concurrent chemoradiotherapy followed by surgery remains the primary treatment option for locally advanced ESCC. In this study, the pCR rate in the Socazolimab + TP group reached 41.4%, a level comparable to results reported for concurrent chemoradiotherapy in the CROSS and NEOCRTEC5010 studies [3, 23]. In addition, complications associated with chemoimmunotherapy seem to be less frequent than those associated with concurrent chemoradiotherapy, indicating that PD-L1 inhibitors are safe and efficient for use in ESCC treatment. For esophageal cancer patients treated with chemoradiotherapy, a recent study demonstrated that T stage was an independent prognostic factor and that a lower stage was related to better prognosis [24]. Moreover, for NSCLC patients receiving neoadjuvant treatment in our previous clinical trial, we acquired similar results: ypT stage instead of ypN stage was significantly correlated with the MPR rate [25], and patients with MPR tended to have a better three-year prognosis [26]. Our analysis revealed that a considerably higher proportion of patients in the Socazolimab + TP group attained the T0 stage than in the Placebo + TP group, suggesting that the anti-PD-L1 antibody may play a key role in tumor downstaging. Due to the short follow-up period, our study has not yet reached the median survival time; however, considering the high pCR rate and the high proportion of patients at the ypT0 stage, we expect that socazolimab plus chemotherapy will promote favorable survival among locally advanced ESCC patients. PD-L1 inhibitor plus chemotherapy may also have several advantages over standard chemoradiotherapy for locally advanced ESCC in neoadjuvant treatment, a potential that may be clarified as the results of several ongoing Phase III trials worldwide—such as KEYSTONE-2, in which pembrolizumab combined with paclitaxel and cisplatin is being evaluated relative to chemoradiotherapy alone—begin to accrue [27].

The pCR rate of the socazolimab plus nab-paclitaxel and cisplatin group was comparable to the rates reported in several Phase II studies of neoadjuvant therapy with PD-1 inhibitors for the treatment of esophageal cancer, indicating comparable efficacy of chemotherapy with PD-1 or PD-L1 inhibitors. For example, in the NIC-ESCC (2019) study, in which 51 ESCC patients were treated with a neoadjuvant therapy program consisting of camrelizumab in combination with nab-paclitaxel and carboplatin, the pCR rate was 39.2% [28]. Similarly, in the SIN-ICE study, which consisted of the administration of sintilimab in combination with nab-paclitaxel and nedaplatin, the pCR rate was 35.5% [29]. Another exploratory treatment for locally advanced ESCC is concurrent chemoradiotherapy coupled with immunotherapy. Several single-arm Phase II trials testing the combination of PD-1 inhibitors and concurrent chemoradiotherapy have reported pCR rates ranging from 46.1–55.6% in ESCC, indicating that this therapy may also be a neoadjuvant treatment strategy worthy of further investigation. Optional strategies for future neoadjuvant treatment of ESCC include chemotherapy, chemoimmunotherapy, concurrent chemoradiotherapy, and concurrent chemoradiotherapy along with immunotherapy. However, Phase III randomized controlled studies are required to determine the most appropriate treatment modality. In addition, each treatment may have a corresponding optimal population, and future research should focus on individualized therapy based on clinical and genetic parameters.

Fan et al., in a single-arm Phase II study, reported that only 13.3% of patients with locally advanced ESCC who underwent neoadjuvant chemotherapy (nab-paclitaxel combined with cisplatin) reached pCR [30]. In contrast, our study reached promising pCR rates perhaps because of more enrolled patients with good health conditions (ECOG 0 score, 84.4% vs. 71.4%), a higher nab-paclitaxel dose (125 mg/m^2^ vs. 100 mg/m^2^), and a longer period of neoadjuvant treatment (four cycles vs. two cycles). In addition, patients in the Placebo + TP group had much higher pCR (27.6%) and MPR (62.1%) rates than patients in the study by Fan et al., which may be one reason why no statistically significant differences in MPR rates were detected between our experimental and control groups.

The median number of cycles of neoadjuvant treatment, proportion of patients receiving reduced dosage, and the overall incidence of Grade 3 or 4 AEs in the Socazolimab + TP group was comparable to that in the Placebo + TP group, although Grade 1 and 2 AEs were somewhat more prevalent in the Socazolimab + TP group than in the Placebo + TP group. There was a higher frequency of hypokalemia and hypoproteinemia in the Socazolimab + TP group than in the Placebo + TP group, possibly because of an increased incidence of gastrointestinal side effects and inadequate supportive care. In addition, irAE incidence was 25.0% in the Socazolimab + TP group, similar to levels (26.0%) reported in a Phase III study of pembrolizumab plus chemotherapy for treatment of esophageal cancer [9] but lower than that (37.0%) for treatment using toripalimab plus chemotherapy [31]. Although the incidence of treatment-related SAEs in the socazolimab plus chemotherapy group was greater than that in the placebo + TP group, it was comparable to the incidence (23.3–30.2%) found in previous studies using PD-1 inhibitor plus chemotherapy approaches [31, 32].

In view of the complexity of esophageal cancer surgery and the possible confounding factors associated with different surgical procedures, we designed strict inclusion criteria, and all cases were uniformly treated by video-assisted thoracoscopic and laparoscopic surgery with three incisions for esophageal cancer (McKeown). Two-field lymph nodes (including the left and right para-laryngeal recurrent nerve lymph nodes) were completely dissected, and the surgical procedure was performed with strict quality control and recorded by video. An enhanced recovery after surgery (ERAS) protocol was applied, and the major surgical complications were counted, excluding cardiac arrhythmias. We harvested more dissected lymph nodes (the maximum numbers of lymph nodes dissected in the experimental and control groups were 69 and 99, respectively) and achieved a lower complication rate than reported in previous studies (3.4% patients with anastomotic leakage in our study compared with 22.0% in the CROSS study), which were closely related to minimally invasive esophagectomy with strict quality control after neoadjuvant chemoimmunotherapy and ERAS implementation.

Our findings demonstrated that the rate of change in PET/CT metabolic parameters (specifically, SUVmax-DR) before and after neoadjuvant treatment had high predictive value for the effectiveness of neoadjuvant treatment for esophageal cancer. SUVmax-DR can be used to accurately identify the majority of patients achieving pCR and MPR, leading to more informed preoperative decisions. Multiple studies have demonstrated that SUVmax-DR and SUVmax after neoadjuvant treatment can predict DFS. We did not analyze the prognostic value of SUVmax-DR because of the limited follow-up time. However, due to the substantial overlap in SUVmax-DR, PET/CT was unable to discriminate pCR from MPR, as was also reported in a previous study [33]. An attempt was made to combine PET/CT with diffusion-weighted magnetic resonance imaging (DW-MRI), the results of which were encouraging, with high rates of sensitivity (90.0%) and specificity (86.0%) [34]. In another study, a model employing PET/CT images was constructed to improve the accuracy of predicting pCR, for which the AUC was 0.81, highlighting the great potential of predictive models based on radiological data [35]. However, inflammatory changes in patients treated with neoadjuvant therapy can also lead to false-positive results because 18F-FDG is not a tumor-specific tracer. Additional research must therefore be conducted when the survival data are mature.ctDNA detection based on a customized assay targeting tumor-specific mutations in plasma cfDNA has shown promising performance in prognostic prediction and disease monitoring in several tumor types, including breast, colorectal and lung cancers [36–38]. In a breast cancer study, patients who remained ctDNA positive after the initiation of chemotherapy were significantly more likely to have residual disease than those who cleared ctDNA [39]. Across cancer types, changes in ctDNA levels from baseline were predictive of benefit from immune checkpoint blockade [40]. We used ctDNA clearance before surgery to predict the efficacy of neoadjuvant immunotherapy in this study. Although there was no significant difference in ctDNA clearance between the experimental and control groups, we discovered that patients in the experimental group had significantly higher ctDNA clearance than those in the control group (36.8% vs. 22.2%). This was similar to the pathological response observed in the two groups. Despite the fact that there was no significant difference, the experimental group outperformed the control group quantitatively.

As a Phase II clinical trial, this study had several limitations. First, there is the possibility of bias due to the limited sample size, and thus, the results need to be further validated in a Phase III trial. Second, follow-up data are lacking, and whether the impressive pCR and MPR rates attained through this therapeutic approach translate into higher DFS or OS rates needs to be explored in future work. Finally, as this was the first study to focus on the combination of socazolimab with nab-paclitaxel and cisplatin as a treatment for esophageal cancer, comparison of our results to those of previous research was problematic because of the lack of preexisting evidence.

## Conclusions

The results of our analysis show that the combination of socazolimab with nab-paclitaxel and cisplatin achieved promising MPR and pCR rates and considerable T-stage downstaging with no concomitant increase in surgical complications, demonstrating the effectiveness and safety of this therapy for treating locally advanced ESCC. Based on these outcomes, we anticipate that combined PD-L1 inhibitor treatment and chemotherapy followed by minimally invasive esophagectomy will become an essential neoadjuvant treatment for locally advanced ESCC.

## Supplementary Information

**Additional file 1.** Methods of ctDNA detection.

**Additional file 2:** **TableS1.** Baseline Characteristics of the Phase Ib Population. **TableS2.** Distribution of Pathologic Stage Groups After Surgery (Phase Ib). **TableS3.** Description of T Stage Changing. **TableS4.** Change of T stages. **TableS5.** Surgery-Related Indicators and Complications. **TableS6.** TRAEs associated with Neoadjuvant Therapy. **TableS7.** TRAEs associated with Neoadjuvant Therapy in Phase Ib (*n*=6). **TableS8.** irAEs associated with Neoadjuvant Therapy in Phase II. **TableS9.** Neoadjuvant Therapy and Perioperative Treatment-related SAEs.

**Additional file 3:** **Fig. S1.** Unique Cases. **Fig. S2.** Dynamic Changes of ctDNA Fraction in 37 Patients during Neoadjuvant Therapy.

**Additional file 4.** CONSORT.

## Data Availability

This clinical trial has been registered on clinicaltrials.gov (registration number NCT04460066) and relevant data can be found on this website.

## Abbreviations

18F-FDG: 18F-fluoro-2-deoxy-d-glucose
AEs: Adverse events
AUC: Area under the curve
C1D1: The first day of the first cycle
C2D1: The first day of the second cycle
C3D1: The first day of the third cycle
C4D1: The first day of the fourth cycle
cfDNA: Cell-free DNA
CI: Confidence interval
CT: Computer tomography
ctDNA: Circulating tumor DNA
DFS: Disease-free survival
DLT: Dose-limiting toxicity
DW-MRI: Diffusion-weighted magnetic resonance imaging
ECOG: Eastern Cooperative Oncology Group
EFS: Event-free survival
ERAS: Enhanced recovery after surgery
ESCC: Esophageal squamous cell carcinoma
HR: Hazard ratio
irAEs: Immune-related AEs
IV: Intravenously
MPR: Major pathological response
NCCN: National Comprehensive Cancer Network
ORR: Overall response rate
OS: Overall survival
pCR: Pathological complete response
PD-1: Programmed cell death protein 1
PD-L1: Programmed cell death ligand 1
PET: Positron emission tomography
preO: Before the operation
ROC: Receiver operating characteristic
SAEs: Severe AEs
SUVmax: Maximum standardized uptake value
SUVmax-DR: Decreased ratio of SUVmax
TP: Nab-paclitaxel + cisplatin
