# Supplementary material for: Comparing a PD-L1 inhibitor plus chemotherapy to chemotherapy alone in neoadjuvant therapy for locally advanced ESCC: a randomized Phase II clinical trial: A randomized clinical trial of neoadjuvant therapy for ESCC
Source: BMC Med. 2023 Mar 8;21:86. doi: 10.1186/s12916-023-02804-y (PMC9993718; doi:10.1186/s12916-023-02804-y)
Supplement: Supplementary file 1 — Additional file 1. Methods of ctDNA detection. [file 12916_2023_2804_MOESM1_ESM.docx]

**Methods of ctDNA detection**

**DNA extraction and library preparation**

Genomic DNA extraction from tumor biopsy tissues and white blood cells was performed using the QIAamp DNA Mini Kit (Qiagen; Hilden, Germany). Subsequently, genomic DNA (250 ng) was subjected to exome sequencing. A gene panel was customized to target 28 mutations in cfDNA (30 ng). Genomic DNA was sheared into small fragments (mean length ~ 200 bp) with the Covaris E220 instrument (Covaris; Woburn, MA, USA). Using the KAPA Hyper Prep Kit (Roche; Basel, Switzerland), the DNA fragments were prepared for genomic libraries through a series of enzymatic steps. End repair, dA tailing, ligation of adaptors, and amplification were performed following standard protocols except for the use of a customized adaptor with barcodes for plasma samples as previously described.

**Whole-exome sequencing and identification of somatic mutations**

The first step was to enrich whole-genome libraries of tumor and matched WBC DNAs for exome regions, which was performed using Agilent SureSelectXT Human All Exon V5 probe and reagents (Agilent; Santa Clara, CA, USA). Subsequently, whole genome sequencing was performed to generate 150 bp paired-end sequences for tumor tissue samples (median coverage 200×) and WBC samples (median coverage 100×) on the Illumina HiSeq X Ten platform (Illumina, San Diego, CA, USA) after removing duplicate molecules. Using Burrows‒Wheeler aligner software (BWA, v0.7.15), the raw reads (FASTQ file) were aligned to the UCSC human reference genome (hg19). Basic processing, marking duplicates, local realignments, and score recalibration were performed using the Genome Analysis Toolkit (GATK, v3.6), Picard (v2.7.1), and Samtools (v1.3.1). The sequencing data from tumor tissue samples were compared with MuTect1 and Strelka to detect candidate somatic mutations, which were then further validated through manual inspection using Integrated Genome Viewer (IGV). A median of 80 mutations were identified in the tumor biopsy tissue samples of each patient.

**cfDNA extraction and library preparation**

cfDNA was extracted directly from plasma using an Apostle MiniMax High Efficiency cfDNA Isolation Kit following the manufacturer’s instructions. The cfDNA libraries were constructed using 30 ng of cfDNA with a KAPA Hyper Prep Kit following standard protocols except for the use of a customized adaptor with barcodes as previously described. The customized adaptor contained sufficient distinct DNA barcodes to identify each original molecule. After adaptor ligation, the DNA fragment was amplified for 10 PCR cycles.

**Customized assay and bioinformatics pipeline**

For each patient, the tumor-specific variants were selected on the basis of their potential to be a driver mutation and the observed mutation frequency in tumor tissue; in particular, somatic single nucleotide variants (SNVs) were preferentially selected. For each mutation, two rounds of nested multiplex PCR were carried out. A target-specific primer and an adapter-matching primer were designed and used to amplify the target regions in the first round. The second round of amplification using one pair of nested primers matching the adapter and the target region further enriched the target region and added the Illumina adapter sequences into the construct.

Customized adaptors contained an eight base-pair random barcode as a unique molecular identifier for each original cfDNA molecule. After removal of the adapter sequences and extraction of the molecular tag sequence, the reads were aligned to the hg19 reference genome. Reads were grouped into families, i.e., unique identifiers (UIDs), based on the same tags and start and end coordinates. A UID family in which more than 80% of reads contained the same mutation as the tumor tissue was considered “effective”. Effective UID families were incorporated into the calculation of mutation frequency and depth with the total number of UID families covering the mutant site. The detailed analysis procedure was described previously.

**Quantification of** **ctDNA fractions**

The cancer cell ratio was estimated based on allele frequency and sequencing depth of somatic mutations in tumor tissue and paired plasma samples. The frequency of each traced somatic mutation in the plasma sample was measured. Finally, cancer cell fractions at the sample level were determined using maximum likelihood estimation.
