# Supplementary material for: Comparing a PD-L1 inhibitor plus chemotherapy to chemotherapy alone in neoadjuvant therapy for locally advanced ESCC: a randomized Phase II clinical trial: A randomized clinical trial of neoadjuvant therapy for ESCC
Source: BMC Med. 2023 Mar 8;21:86. doi: 10.1186/s12916-023-02804-y (PMC9993718; doi:10.1186/s12916-023-02804-y)
Supplement: Supplementary file 2 — Additional file 2: TableS1. Baseline Characteristics of the Phase Ib Population. TableS2. Distribution of Pathologic Stage Groups After Surgery (Phase Ib). TableS3. Description of T Stage Changing. TableS4. Change of T stages. TableS5. Surgery-Related Indicators and Complications. TableS6. TRAEs associated with Neoadjuvant Therapy. TableS7. TRAEs associated with Neoadjuvant Therapy in Phase Ib (n=6). TableS8. irAEs associated with Neoadjuvant Therapy in Phase II. TableS9. Neoadjuvant Therapy and Perioperative Treatment-related SAEs. [file 12916_2023_2804_MOESM2_ESM.docx]

**Additional File 2: Tables**

**Additional File 2: Table. S1.** Baseline Characteristics of the Phase Ib Population

|  | Phase Ib (n=6) |
| --- | --- |
| Age, years |  |
| Median (range) | 63.0 (60 - 70) |
| Sex |  |
| Male | 5 (83.3) |
| Female | 1 (16.7) |
| BMI, kg/m2 |  |
| Median (range) | 22.1 (21.6 - 27.4) |
| ECOG PS |  |
| 0 | 4 (66.7) |
| 1 | 2 (33.3) |
| Tumor location |  |
| Proximal third | 1 (16.7) |
| Middle third | 2 (33.3) |
| Distal third | 3 (50.0) |
| Clinical T stage |  |
| cT2 | 0 (0.0) |
| cT3 | 6 (100.0) |
| cT4a | 0 (0.0) |
| Clinical N stage |  |
| cN0 | 3 (50.0) |
| cN1 | 1 (16.7) |
| cN2 | 1 (16.7) |
| cN3 | 1 (16.7) |
| Clinical stage |  |
| II | 3 (50.0) |
| III | 2 (33.3) |
| IVA | 1 (16.7) |

NOTE. Data are presented as No. (%).

Abbreviations: BMI, body mass index; ECOG, Eastern Cooperative Oncology Group performance status.

**Additional File 2: Table. S2.** Distribution of Pathologic Stage Groups After Surgery (Phase Ib)

|  | Phase Ib (n=4) |
| --- | --- |
| Pathologic T Stage |  |
| T0 | 1 (25.0) |
| Tis/T1 | 3 (75.0) |
| Pathologic N Stage |  |
| N0 | 3 (75.0) |
| N1 | 1 (25.0) |
| Postoperative Stage |  |
| I | 3 (75.0) |
| II | 1 (25.0) |

NOTE. Data are presented as No. (%).

**Additional File 2: Table. S3.** Description of T Stage Changing

|  | Socazolimab + TP (n = 29) | Placebo + TP  (n = 29) | *P* |
| --- | --- | --- | --- |
| Downstaging | 19 (65.5) | 18 (62.1) | 0.784 |
| T4 to T0 | 1 (5.3) | 0 (0.0) |  |
| T3 to T0 | 7 (36.8) | 0 (0.0) |  |
| T2 to T0 | 3 (15.8) | 1 (5.6) |  |
| T3 to Tis/T1 | 5 (26.3) | 13 (72.2) |  |
| T3 to T2 | 3 (15.8) | 4 (22.2) |  |
| Unchange | 10 (34.5) | 9 (31.0) | 0.779 |
| Upstaging | 0 (0.0) | 2 (6.9) | 0.469 |
| T2 to T3 | 0 (0.0) | 2 (6.9) |  |

NOTE. Data are presented as No. (%).

Abbreviations: TP, nab-paclitaxel + cisplatin.

**Additional File 2: Table. S4.** Change of T stages

|  | Socazolimab + TP  (n = 19) | Placebo + TP  (n = 18) | *P* |
| --- | --- | --- | --- |
| Downstages |  |  | 0.015 |
| 4 | 1 (5.3) | 0 (0.0) |  |
| 3 | 7 (36.8) | 0 (0.0) |  |
| 2 | 8 (42.1) | 14 (77.8) |  |
| 1 | 3 (15.8) | 4 (22.2) |  |
| Downstages |  |  | 0.003 |
| 3-4 | 8 (42.1) | 0 (0.0) |  |
| 1-2 | 11 (57.9) | 18 (100.0) |  |
| Downstage to |  |  | 0.002 |
| T0 | 11 (57.9) | 1 (5.6) |  |
| Tis/T1 | 5 (26.3) | 13 (72.2) |  |
| T2 | 3 (15.8) | 4 (22.2) |  |
| Downstage to |  |  | 0.001 |
| T0 | 11 (57.9) | 1 (5.6) |  |
| Tis/T1-2 | 8 (42.1) | 17 (94.4) |  |

NOTE. Data are presented as No. (%).

Abbreviations: TP, nab-paclitaxel + cisplatin.

**Additional File 2: Table. S5.** Surgery-Related Indicators and Complications

|  | Socazolimab + TP  (n = 29) | Placebo + TP  (n = 29) | *P* |
| --- | --- | --- | --- |
| Length of hospital stay (days) |  |  |  |
| Median (range) | 13 (8 - 36) | 11 (7 - 25) | 0.461 |
| Duration of surgery (min) |  |  |  |
| Median (range) | 240 (160 - 563) | 230 (151 - 396) | 0.447 |
| Bleeding volume (ml) |  |  |  |
| Median (range) | 50 (50 - 1800) | 50 (20 - 800) | 0.291 |
| Number of lymph node dissections (pcs) |  |  |  |
| Median (range) | 44 (10 - 69) | 35 (16 - 99) | 0.312 |
| Time from last neoadjuvant therapy administration to surgery (days) |  |  |  |
| Median (range) | 47 (28 - 127) | 45 (31 - 67) | 0.196 |
| Major Surgical Complications |  |  |  |
| Anastomosis fistula | 1^*^ (3. 4) | 0 (0.0) |  |
| Bile duct obstruction | 1^*^ (3. 4) | 0 (0.0) |  |
| Pneumonia | 1 (3. 4) | 1^#^ (3.4) |  |
| Pneumothorax | 0 (0.0) | 1 (3.4) |  |
| Reoperation | 0 (0.0) | 0 (0.0) |  |
| Perioperative death | 0 (0.0) | 1^#^ (3.4) |  |

NOTE. Data are presented as No. (%).

Abbreviations: TP, nab-paclitaxel + cisplatin.

The patients marked * and # in the table represent the same patient.

**Additional File 2: Table. S6.** TRAEs associated with Neoadjuvant Therapy

| AE | Socazolimab + TP  (n = 32) | | Placebo + TP  (n = 32) | | |
| --- | --- | --- | --- | --- | --- |
|  | all | ≥ level 3 | | all | ≥ level 3 |
| Total | 32 (100.0) | 21 (65.6) | | 32 (100.0) | 20 (62.5) |
| Decreased neutrophil count | 25 (78.1) | 19 (59.4) | | 25 (78.1) | 18 (56.3) |
| Decreased white blood cell count | 25 (78.1) | 14 (43.8) | | 22 (68.8) | 8 (25.0) |
| Anaemia | 32 (100.0) | 4 (12.5) | | 27 (84.4) | 2 (6.3) |
| Febrile neutropenia | 2 (6.3) | 2 (6.3) | | 0 (0.0) | 0 (0.0) |
| Decreased platelet count | 32 (100.0) | 4 (12.5) | | 27 (84.4) | 2 (6.3) |
| Hyponatremia | 11 (34.4) | 2 (6.3) | | 3 (9.4) | 1 (3.1) |
| Hypokalemia | 11 (34.4) | 6 (18.8) | | 1 (3.1) | 0 (0.0) |
| Hypoalbuminemia | 8 (25.0) | 0 (0.0) | | 4 (12.5) | 0 (0.0) |
| Elevated serum creatinine | 6 (18.8) | 1 (3.1) | | 7 (21.9) | 0 (0.0) |
| Elevated aspartate aminotransferase | 4 (12.5) | 0 (0.0) | | 3 (9.4) | 1 (3.1) |
| Elevated alanine aminotransferase | 2 (6.3) | 0 (0.0) | | 4 (12.5) | 0 (0.0) |
| Elevated blood bilirubin | 1 (3.1) | 0 (0.0) | | 2 (6.3) | 0 (0.0) |
| Fatigue | 12 (37.5) | 0 (0.0) | | 8 (25.0) | 0 (0.0) |
| Loss of appetite | 17 (53.1) | 1 (3.1) | | 9 (28.1) | 0 (0.0) |
| Sickness | 13 (40.6) | 0 (0.0) | | 10 (31.3) | 0 (0.0) |
| Vomiting | 6 (18.8) | 0 (0.0) | | 7 (21.9) | 0 (0.0) |
| Diarrhea | 6 (18.8) | 1 (3.1) | | 2 (6.3) | 0 (0.0) |
| Hair loss | 10 (31.3) | 0 (0.0) | | 9 (28.1) | 0 (0.0) |
| Rash | 4 (12.5) | 0 (0.0) | | 0 (0.0) | 0 (0.0) |
| Fever | 3 (9.4) | 0 (0.0) | | 2 (6.3) | 0 (0.0) |
| Infectious pneumonia | 2 (6.3) | 0 (0.0) | | 2 (6.3) | 2 (6.3) |
| Urinary tract infection | 2 (6.3) | 1 (3.1) | | 0 (0.0) | 0 (0.0) |
| Joint pain | 1 (3.1) | 0 (0.0) | | 3 (9.4) | 0 (0.0) |
| Peripheral sensory neuropathy | 3 (9.4) | 0 (0.0) | | 0 (0.0) | 0 (0.0) |

NOTE. Data are presented as No. (%).

Abbreviations: TRAE, treatment-related adverse events; AE, adverse events; TP, nab-paclitaxel + cisplatin.

**Additional File 2: Table. S7.** TRAEs associated with Neoadjuvant Therapy in Phase Ib (n=6)

| AE | all | ≥ level 3 |
| --- | --- | --- |
| Total | 6 (100.0) | 6 (100.0) |
| Decreased white blood cell count | 6 (100.0) | 2 (33.3) |
| Decreased neutrophil count | 6 (100.0) | 6 (100.0) |
| Anemia | 6 (100.0) | 1 (16.7) |
| Elevated aspartate aminotransferase | 3 (50.0) | 0 (0.0) |
| Elevated alanine aminotransferase | 1 (16.7) | 0 (0.0) |
| Hypokalemia | 1 (16.7) | 0 (0.0) |
| Loss of appetite | 4 (66.7) | 0 (0.0) |
| Nausea | 2 (33.3) | 0 (0.0) |
| Vomiting | 1 (16.7) | 0 (0.0) |
| Fatigue | 2 (33.3) | 0 (0.0) |
| Fever | 1 (16.7) | 0 (0.0) |
| Hair loss | 4 (66.7) | 0 (0.0) |
| Rash* | 1 (16.7) | 0 (0.0) |
| Immune pneumonitis* | 1 (16.7) | 1 (16.7) |
| Hypothyroidism* | 2 (33.3) | 0 (0.0) |
| Neurotoxicity | 1 (16.7) | 1 (16.7) |
| Myocarditis* | 1 (16.7) | 1 (16.7) |

NOTE. Data are presented as No. (%).

Abbreviations: TRAE, treatment-related adverse events; AE, adverse events.

**Additional File 2: Table. S8.** irAEs associated with Neoadjuvant Therapy in Phase II

| irAE | Socazolimab + TP  (n = 32) | |
| --- | --- | --- |
|  | all | ≥ grade 3 |
| Total | 8 (25.0) | 1 (3.1) |
| Elevated thyroxine | 2 (6.3) | 0 (0.0) |
| Decreased neutrophil count | 1 (3.1) | 0 (0.0) |
| Decreased white blood cell count | 0 (0.0) | 0 (0.0) |
| Elevated alanine aminotransferase | 0 (0.0) | 0 (0.0) |
| Decreased lymphocyte count | 1 (3.1) | 0 (0.0) |
| Elevated aspartate aminotransferase | 0 (0.0) | 0 (0.0) |
| Elevated thyroid stimulating hormone | 0 (0.0) | 0 (0.0) |
| Elevated blood bilirubin | 0 (0.0) | 0 (0.0) |
| Elevated serum creatinine | 1 (3.1) | 0 (0.0) |
| Elevated blood urea | 1 (3.1) | 0 (0.0) |
| Decreased platelet count | 1 (3.1) | 0 (0.0) |
| Rash | 1 (3.1) | 0 (0.0) |
| Pruritus | 1 (3.1) | 0 (0.0) |
| hypoalbuminemia | 0 (0.0) | 0 (0.0) |
| Vomiting | 1 (3.1) | 0 (0.0) |
| Arrhythmia | 1 (3.1) | 0 (0.0) |

NOTE. Data are presented as No. (%).

Abbreviations: irAE, immune--related adverse events; TP, nab-paclitaxel + cisplatin.

The p-value is > 0.05.

**Additional File 2: Table. S9.** Neoadjuvant Therapy and Perioperative Treatment-related SAEs

|  | Socazolimab + TP (n = 32) | Placebo + TP  (n = 32) |
| --- | --- | --- |
| Total | 9 (28.1) | 4 (12.5) |
| Infectious pneumonia | 1 (3. 1) | 2 (6.3) |
| Immune pneumonitis | 1 (3.1) | 0 (0.0) |
| Pneumothorax | 0 (0.0) | 1 (3.1) |
| Anastomotic fistula | 1 (3.1) | 0 (0.0) |
| Urinary Tract Infection/Acute Kidney Injury | 1 (3.1) | 0 (0.0) |
| Neutropenia | 3 (9.4) | 0 (0.0) |
| Febrile neutropenia | 1 (3.1) | 0 (0.0) |
| Decreased platelet count | 1 (3. 1) | 0 (0.0) |
| Anemia | 1 (3.1) | 0 (0.0) |
| Intestinal obstruction | 1 (3.1) | 1 (3.1) |
| Cholecystitis | 1 (3.1) | 0 (0.0) |
| Diarrhea | 1 (3.1) | 0 (0.0) |
| Hypokalemia | 1 (3.1) | 0 (0.0) |
| Hyponatremia | 1 (3.1) | 0 (0.0) |
| Arrhythmia | 0 (0.0) | 1 (3.1) |

NOTE. Data are presented as No. (%).

Abbreviations: SAE, severe adverse events; TP, nab-paclitaxel + cisplatin.
