# Supplementary material for: Comparing a PD-L1 inhibitor plus chemotherapy to chemotherapy alone in neoadjuvant therapy for locally advanced ESCC: a randomized Phase II clinical trial: A randomized clinical trial of neoadjuvant therapy for ESCC
Source: BMC Med. 2023 Mar 8;21:86. doi: 10.1186/s12916-023-02804-y (PMC9993718; doi:10.1186/s12916-023-02804-y)
Supplement: Supplementary file 3 — Additional file 3: Fig. S1. Unique Cases. Fig. S2. Dynamic Changes of ctDNA Fraction in 37 Patients during Neoadjuvant Therapy. [file 12916_2023_2804_MOESM3_ESM.docx]

**Additional File 3: Figures**


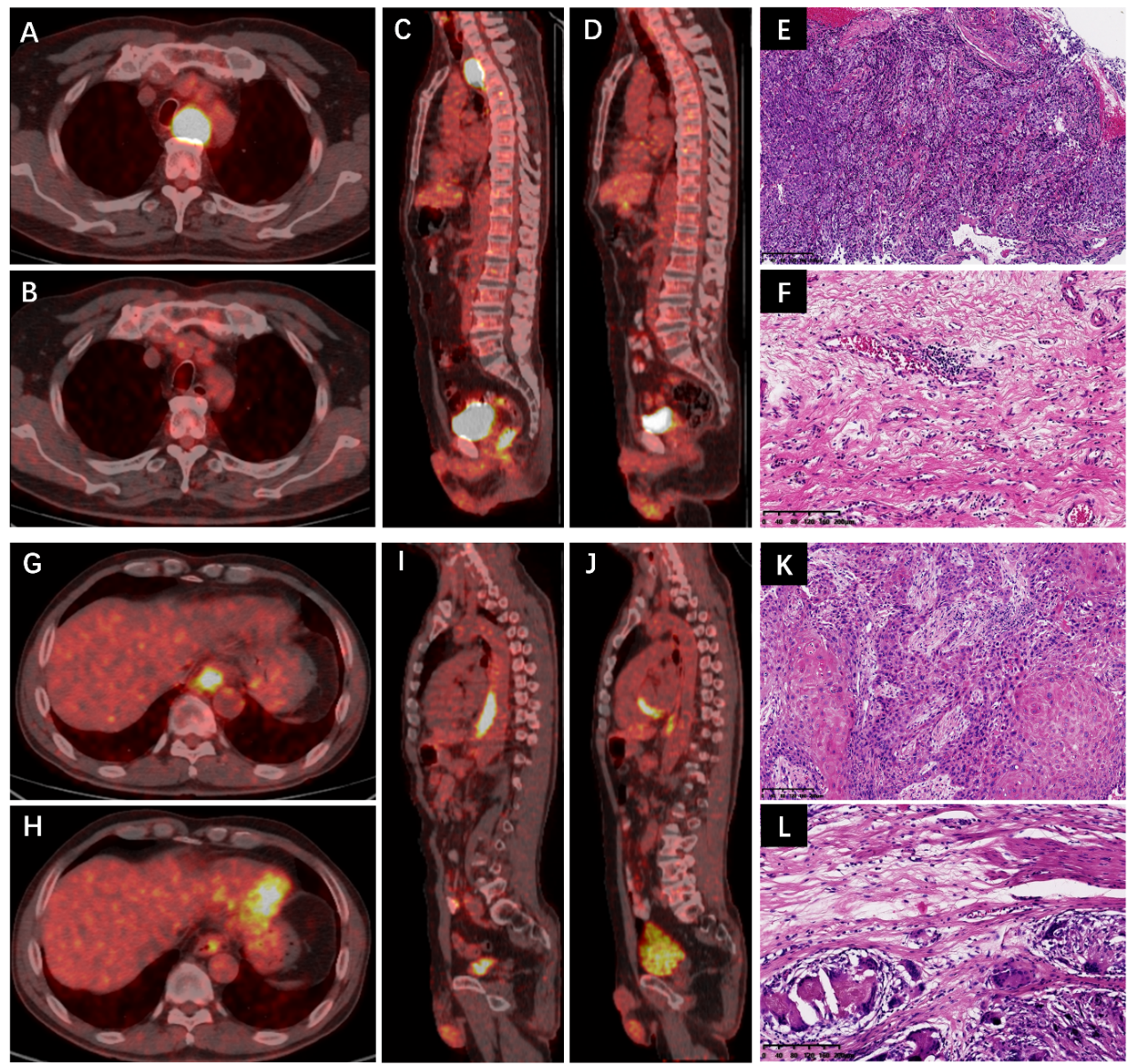


**Additional File 3: Fig. S1**. Unique cases.

(1) A 71-year-old male with upper low-grade esophageal squamous cell carcinoma in the Socazolimab + TP group (four cycles of chemoimmunotherapy) with a surgical pathology evaluation of pCR. Shown here are images for the baseline PET/CT axial fusion (A) and sagittal fusion (C), with SUVmax 20.5 for the primary lesion. The pathological images (E, 100X) in tumor bed of the samples by gastroscopic biopsy at baseline before neoadjuvant therapy are presented. The preoperative PET/CT axial fusion (B) and sagittal fusion (D) were presented, and evaluated as complete metabolic remission (CMR) according to PERCIST criteria. The pathological images (F, 100X) in tumor bed of the surgical samples are presented. (2) A 67-year-old male, highly differentiated squamous cell carcinoma of the lower mid-thoracic esophagus, in the Placebo + TP group (four cycles of chemotherapy), surgical pathology evaluated as pCR, but multiple polypoid hyperplastic nodules were visible on the surface. The baseline PET/CT axial fusion images (G) and sagittal fusion images (I) were presented, with SUVmax 17.7 for the primary lesion. The pathological images (K, 100X) in tumor bed of the samples by gastroscopic biopsy at baseline before neoadjuvant therapy are presented. The preoperative PET/CT axial fusion images (H) and sagittal fusion images (J) were presented, with localized focal hypermetabolism and SUVmax 4.2, and evaluated as partial metabolic remission (PMR) according to PERCIST criteria. The pathological images (L, 100X) in tumor bed of the surgical samples are presented.


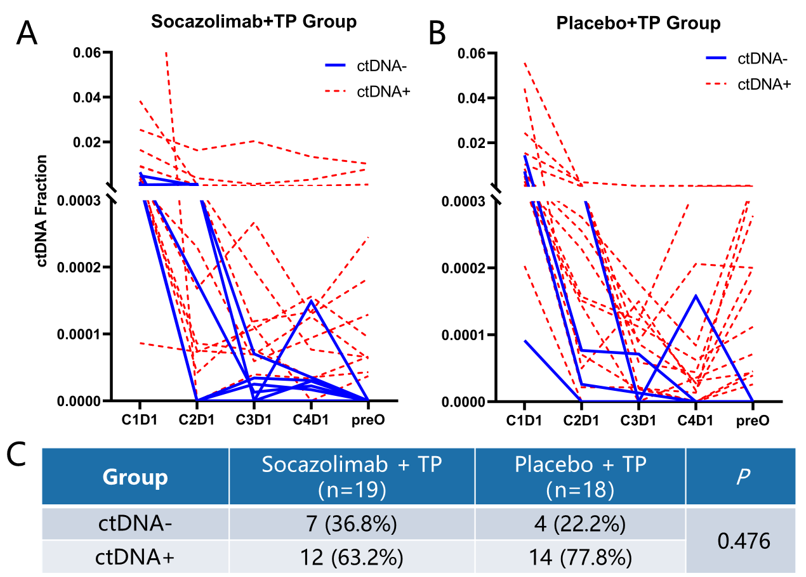


**Additional File 3: Fig. S2**. Dynamic changes of ctDNA fraction in 37 patients during neoadjuvant therapy.

A. ctDNA fraction of 19 patients in the Socazolimab + TP group. B. ctDNA fraction of 18 patients in the Placebo + TP group. The abscissa denotes the time of blood collection. For example, C1D1 denotes the time of blood collection on the first day of the first cycle of neoadjuvant therapy prior to drug therapy, and so on. PreO is the time point at which blood was collected at the end of four cycles of neoadjuvant therapy and one day before surgery. ctDNA- represents the ctDNA clearance at preO. ctDNA+ represents the patients whose ctDNA were not cleared at preO. C. Summary table of ctDNA results of 37 patients at preO. Abbreviations: ctDNA, circulating tumor DNA; TP, nab-paclitaxel + cisplatin; PreO, before operation.
